# Immunity and nutrition: from research to clinical practice

**DOI:** 10.1186/1824-7288-40-S2-A31

**Published:** 2014-10-09

**Authors:** Francesco Tandoi, Francesco Pellegrini, Massimo Agosti

**Affiliations:** 1 Neonatology & NICU Department, “F. Del Ponte” Hospital, 21100 Varese, Italy

## 

Human immune system is a complex and efficient defence system, consisting of an integrated set of cells and chemical mediators, which protects from external insults (chemical, traumatic or infectious), in addition to a series of defensive “modular” responses to protect and modulate immune response inside our body.

It is extremely difficult to determine what might be the influence of diet on this system. This is true not only for adults, but can be extended also to every phase of life.

To express the correlation between nutrition and immunity in the early stages of an individual's life it is absolutely essential the knowledge of both subjects, immunity and nutrition, to justify any intervention carried out in this early stage of development. It assumes even a greater value if we talk about newborn infants –preterms especially- maximum expression of an immature system, conditioned in all its forms by any external intervention.

Theoretically, the limited opportunities in neonatal nutrition (breast milk or formula) make easier to report specific nutritional effects on any of its functions although in reality it is much more complex.

In newborn a nutritional "programming" can already be affected by prenatal interferences. In order to characterize the correlation between nutritional interventions and development of immunity some interesting clinical trials in developing countries are in progress (eg, ENID: Early Nutrition and Immune Development, The Gambia); this study wants to investigate if some interventions, conducted in pregnant woman and in the early stages of childhood, can modify parameters such as anthropometry at birth and during the first months of life, morbidity, thymus dimension and some biochemical immune responses [1].

About newborn infants, other defence mechanism must be considered: 1) the system of maternal IgG antibodies that fetus receives, via the placenta; 2) the system of breast-milk proteins: secretory IgA antibodies which bind the microbes on the infant’s mucosal membranes; lactoferrin which destroy microbes and reduce inflammatory responses, non-absorbed milk oligosaccharides which block attachment of microbes to the infant’s mucosae; other numerous additional proteins in the milk as the anti-secretory factor, which is anti-inflammatory, preventing diarrhoea in infants; 4) the system of micronutrients as zinc, vitamin A, B, C, iron and cytokines [2,3].

In any case breastfeeding and a good nutritional status are essential to give a functionally correct amount of these nutrients. Even the development of adapted formulas is directed to clarify functional actions of nutrients, including the improvement of immune response [4].
